# Salinity Tolerance of Two Potato Cultivars (*Solanum tuberosum*) Correlates With Differences in Vacuolar Transport Activity

**DOI:** 10.3389/fpls.2018.00737

**Published:** 2018-06-05

**Authors:** Rinse Jaarsma, Albertus H. de Boer

**Affiliations:** Faculty of Earth and Life Sciences, Vrije Universiteit Amsterdam Amsterdam, Netherlands

**Keywords:** potato, vacuolar proton transport, Na^+^/H^+^ exchange, Na^+^/H^+^ antiporters, vacuolar H^+^-ATPase, vacuolar H^+^-PPase, tonoplast vesicles, salt tolerance

## Abstract

Potato is an important cultivated crop species and since it is moderately salt sensitive there is a need to develop more salt tolerant cultivars. A high activity of Na^+^ transport across the tonoplast in exchange for H^+^ is essential to reduce Na^+^ toxicity. The proton motive force (PMF) generated by the V-H^+^-ATPase and the V-H^+^-PPase energizes the Na^+^(K^+^)/H^+^ antiport. We compared the activity, gene expression, and protein levels of the vacuolar proton pumps and the Na^+^/H^+^ antiporters in two potato cultivars (*Solanum tuberosum*) contrasting in their salt tolerance (cv. Desiree; tolerant and Mozart; sensitive) grown at 0 and 60 mM NaCl. Tonoplast-enriched vesicles were used to study the pump activity and protein levels of the V-H^+^-ATPase and the V-H^+^-PPase and the activity of the Na^+^/H^+^ antiporter. Although salt stress reduced the V-H^+^-ATPase and the V-H^+^-PPase activity in both cultivars, the decline in H^+^ pump activity was more severe in the salt-sensitive cultivar Mozart. After salt treatment, protein amounts of the vacuolar H^+^ pumps decreased in Mozart but remained unchanged in the cultivar Desiree. Decreased protein amounts of the V-H^+^-PPase found in Mozart may explain the reduced V-H^+^-PPase activity found for Mozart after salt stress. Under non-stress conditions, protein amounts of V-H^+^-PPase were equal in both cultivars while the V-H^+^-PPase activity was already twice as high and remained higher after salt treatment in the cultivar Desiree as compared to Mozart. This cultivar-dependent V-H^+^-PPase activity may explain the higher salt tolerance of Desiree. Moreover, combined with reduced vacuolar H^+^ pump activity, Mozart showed a lower Na^+^/H^+^ exchange activity and the *K*_m_ for Na^+^ is at least twofold lower in tonoplast vesicles from Desiree, what suggests that NHXs from Desiree have a higher affinity for Na^+^ as compared to Mozart. From these results, we conclude that the higher capacity in combination with the higher affinity for Na^+^ uptake can be an important factor to explain the differences in salt tolerance of these two potato cultivars.

## Introduction

Salinity is a serious threat to agricultural production of many crops. Potato is an important staple food in the human diet in many countries worldwide and the trend of potato production is toward more hectares in warmer and dryer climates (Levy and Veilleux, 2007). Soil salinization, often the result of poor irrigation practices, is one of the factors that limit potato production. Based on specific criteria, potato was found to be moderately sensitive to salinity and considering the importance of potato as a staple food, relatively few studies address the physiological, biochemical, and molecular responses that take place during salt stress in potato (Hmida-Sayari et al., 2005; Aghaei et al., 2009; Legay et al., 2009; Batelli et al., 2012; Jaarsma et al., 2013).

Current insights show that plants have developed many mechanisms to tolerate salt stress and three categories of adaptation can be distinguished, namely, osmotic tolerance, ion exclusion, and tissue tolerance (Munns and Tester, 2008; Roy et al., 2014). Roy et al. (2014) describe ion exclusion as an important mechanism to prevent Na^+^ accumulation into shoots and leaves. However, if Na^+^ travels into cells of leaves, efficient removal by compartmentalizing Na^+^ in vacuoles prevents detrimental effects on cellular processes (Munns and Tester, 2008) such as premature senescence, already occurring over several days to weeks after salt stress (Roy et al., 2014). In a previous study, we found a strong premature senescence response in the leaves of the potato cultivar Mozart combined with relatively high Na^+^ leaf accumulation, while the potato cultivar Desiree accumulated less Na^+^ in leaves and did not show a senescence response after 7 days of salt treatment and we concluded that Desiree was salt tolerant and that Mozart was salt sensitive (Jaarsma et al., 2013). In the same study, we hypothesized that the vacuolar sequestration capacity of Na^+^ in leaves of Mozart was lower than that of the more salt tolerant potato cultivar.

In vacuoles, Na^+^ serves as an inorganic osmoticum to maintain turgor (Shabala and Mackay, 2011). Na^+^ may enter vacuoles by the activity of Na^+^/H^+^ antiporters energized by a proton motive force (PMF) established by vacuolar proton pumps. So far, there is still debate as to which gene or protein facilitates the Na^+^/H^+^ antiport activity; however, many studies suggest a role for members of the NHX family (Munns and Tester, 2008; Rodríguez-Rosales et al., 2009; Pardo and Rubio, 2011), although AtNHX1 also catalyzes K^+^/H^+^ antiport (Jiang et al., 2010) and SlNHX2 from tomato (*Solanum lycopersicum*) was found to catalyze solely K^+^/H^+^ antiport (Rodríguez-Rosales et al., 2008). Hence, dual functions for NHX exchangers have been proposed (Yamaguchi et al., 2005; Jiang et al., 2010), and NHX isoforms also play significant roles in other cellular processes, such as membrane trafficking, pH- and ion homeostasis, stomatal regulation, cell turgor and growth and development (Bassil et al., 2011; Eckardt and Berkowitz, 2011; Barragan et al., 2012; Bassil and Blumwald, 2014; Reguera et al., 2015). Many studies showed improved salt tolerance and increased accumulation of Na^+^ or K^+^ when *NHX* was expressed in a variety of plant species (Agarwal et al., 2012). However, expression of *AtNHX1* in barley did not improve plant performance during saline conditions (Adem et al., 2015) and it was suggested before that efficient vacuolar Na^+^ sequestration also depends on the permeability of slow- and fast-activating vacuolar channels (Bonales-Alatorre et al., 2013).

To transport ions into the vacuoles, an electrochemical H^+^ gradient across the tonoplast has to be established by V-H^+^-ATPases and/or V-H^+^-PPases, using ATP and PPi as a substrate, respectively. Transcript levels of *V-H^+^-ATPase* genes were only slightly induced in barley after salt stress (Fukuda et al., 2004) and in the woody plant *Broussonetia papyrifera*, transcript levels of several V-H^+^-ATPase subunits increased upon salt treatment in root and leaf tissues (Zhang et al., 2012). Moreover, transgenic barley plants expressing the V-H^+^-ATPase subunit C from Arabidopsis and Arabidopsis plants expressing subunits from wheat were found to be more salt tolerant (He et al., 2014; Adem et al., 2017). However, Arabidopsis mutant analysis showed that other roles for the V-H^+^-ATPase are also found, like efficient nutrient storage (Krebs et al., 2010) and membrane trafficking, depending on their cellular localization (Schumacher and Krebs, 2010).

The wheat vacuolar proton-pumping pyrophosphatase (H^+^-PPase) genes (*TaVP1* and *TaVP2*) showed induced expression in response to salinity (Wang et al., 2009) and were shown to have major roles in abiotic stress among other functions, such as maintaining cellular PPi homeostasis, heterotrophic growth, increased auxin transport, and sucrose transport from source to sink tissues (Schilling et al., 2017). In the context of salt tolerance, transgenic plants expressing *H^+^-PPase* accumulated more Na^+^ in leaves or shoots and had higher V-H^+^-PPase activity compared to wild-type plants (Gaxiola et al., 2001), while other transgenic plants expressing *H^+^-PPase* did not show higher Na^+^ accumulation but may still compartmentalize more Na^+^ in their vacuoles (Schilling et al., 2014). In addition, Queiros et al. (2009) provided evidence that a more efficient Na^+^ sequestration mechanism improves salt tolerance in potato cells (Queiros et al., 2009). They adapted calli to 150 mM NaCl and the selected NaCl-tolerant calli lines showed higher ATP- and PPi-dependent H^+^ transport and higher Na^+^/H^+^ antiport activity as compared to control cells. However, the vacuolar Na^+^ sequestration system and its response to salinity stress in commercial potato cultivars have not been studied yet.

In this study, we compared Mozart and Desiree for the salt-induced changes in activity and expression of vacuolar H^+^-pumps and vacuolar antiporters isolated from leaves. The main conclusion is that the *V*_max_ of both the V-H^+^-ATPase and the V-H^+^-PPase were reduced by salt in both Mozart and Desiree but the reduction was larger in Mozart as compared to Desiree. Furthermore, after salt treatment, the amount of both V-H^+^-ATPase and V-H^+^-PPase proteins was reduced in Mozart but not in Desiree. In addition, the Na^+^/H^+^ exchange system in the tolerant cultivar Desiree works more efficient as compared to the salt-sensitive cultivar Mozart.

## Materials and Methods

### Plant Material and Growth Conditions

Two-week-old cuttings of two potato (*Solanum tuberosum*) cultivars Desiree and Mozart were planted in 4 L pots containing ½ strength Hoagland solution and was refreshed every fourth day. Each pot contained four cuttings of the same cultivar held by a rockwool plug embedded in styrofoam in the lid of the pot. Plants were maintained in a growth chamber under a 16/8 h light/dark photoperiod, 24°C/15°C day/night temperature and 70% relative humidity. After 3 weeks, plants were subjected to two treatments: 0 mM NaCl and 60 mM NaCl. The salt treatment was applied two times, at the first and third day, respectively, and after 5 days the plants were harvested for further analysis. Both treatments were done with three or four independent biological replicates as indicated in the figures. The 0 mM NaCl treatment was normal ½ strength Hoagland solution and the 60 mM NaCl treatment was ½ strength Hoagland solution supplemented with 60 mM NaCl. Each pot containing four cuttings was considered a biological replicate.

### Ion Analysis, Fresh/Dry Weight Ratio and Leaf Sap Osmolality

At the end of each experiment, a small part of fresh leaves (∼2 g) was collected from 10 leaves. The leaves were collected from all parts of the plant to obtain a representative mix of old and young leaves in a similar way for all plants. Subsequently, the leaves were weighed to determine fresh weight (FW), before snap freezing in liquid nitrogen. To collect the leaf sap, each sample was ground for 30 s at room temperature and centrifuged at 15,000 *g* for 5 min. Eight microliters of the supernatant was used to estimate the osmolality with an osmometer (Wescor 5500, Logan, UT, United States). Determination of Na^+^ and K^+^ was done as described before (Jaarsma et al., 2013). In short, fresh material was dried at 70°C for 24 h and 100 mg dried plant material was extracted by 1 h boiling in 5 ml MilliQ. The solution was filtered through 0.2 μm filters (Whatman, England) and Na^+^ and K^+^ concentrations in the filtrate were analyzed using high-performance liquid chromatography (HPLC, Shimadzu, Japan). The HPLC system was equipped with a ø 4.6 mm × 125 mm Shodex IC YS-50 column (Showa Denko). As an eluent, 4.0 mM methane sulfonic acid was used in HPLC-graded H_2_O (J.T. Baker, Netherlands) with a flow rate of 1 ml/min. Final ion concentrations in the filtrate were calculated according to a calibration curve.

### Candidate Genes and Gene Expression Analysis

Unigenes or genomic sequences with homology to *SlVP1* and *SlVP2* and *SlNHX1–SlNHX4* were identified in potato by querying Potato databases^1^^,^^2^. Homology to tomato was determined at the nucleotide level and amino acid level. Potato unigenes from the SOL genomics database and the sequences from the Potato Genome Sequencing Consortium were designated *StNHX1–StNHX4* according to *SlNHXs* and *StVP1* and *StVP2* according to *SlVP1* and *SlVP2*. Unigenes were used to design primers with optimum settings of 40–60% GC content, 20 nt in length, primer Tm of 60°C and an amplicon size of 100 bp. Designed primers were used in blast searches against the previously mentioned databases and only the primers that showed no significant matches other than the unigenes they were designed for were further tested. Subsequently, transcript levels were determined by RT-PCR and qPCR using cDNA from potato plants subjected to 0 and 60 mM NaCl. Those gene-specific primer pairs that resulted in a sharp band of the expected amplicon size on agarose gels and showed a sharp peak after software analysis were chosen for qPCR analysis and are listed in **Table 2**.

RNA was isolated from leaves using the Nucleospin RNeasy plant mini kit (Qiagen, Leusden, Netherlands) and all samples were treated with DNase. Tissue was ground in liquid nitrogen using a mortar and pestle. Approximately 100 mg of ground tissue was used for RNA isolation according to the manufacturer’s instructions. cDNA was synthesized from 1 to 4 μg RNA using Superscript II (Invitrogen) in a 20-μl reaction volume containing 5 μM oligo dT (T15) primer according to the manufacturer’s protocol. qPCRs contained 100 ng cDNA, 0.5 pmol of each primer (see candidate genes) and 2× Sybr Green PCR buffer (Bio-Rad, Hercules, CA, United States). The program for qPCR was set to 2 min at 50°C, 5 min at 95°C, continued by 40 cycles of 95°C for 30 s and 30 s at 60°C and a melting curve analysis. qPCRs were performed in the CHROMO4 MJ research PTC200 (Bio-Rad, Hercules, CA, United States). Data were analyzed according to the qgene 96 program (Muller et al., 2002). The melting curves showed only one sharp peak at the expected melting temperature confirming that the amplification was gene-specific. The potato elongation factor *ef1α* gene (AB061263, forward primer 5′-3′ATTGGAAACGGATATGCTCCA, reverse primer 5′-3′TCCTTACCTGAACGCCTGTCA) was used as a reference gene since this gene was shown to be stable during salt stress (Nicot et al., 2005).

### SDS-PAGE and Immunoblotting

Vacuolar membrane proteins (10 μg) were separated on SDS-PAGE [10% (w/v) polyacrylamide]. Prior to electrophoresis, samples were heated at 70°C for 10 min, in 50 mM Tris–HCl, pH 6.8, 2% (w/v) sodium dodecyl sulphate (SDS), 5% (v/v) β-mercaptoethanol, and 10% (v/v) glycerol. Subsequently, the gels were stained with Coomassie Brilliant Blue [0.125% (w/v) in 50% (v/v) methanol]/10% (v/v) acetic acid for 30 min and destained in 5% (v/v) methanol/7.5% (v/v) acetic acid overnight. Stained gels were scanned into greyscale and the optical density was determined using Scion Image. After scanning, proteins in the gels were electro-transferred onto a nitrocellulose membrane (Schleicher and Schuel, Dassel, Germany) in a transfer buffer containing 50 mM Trizma, 380 mM glycine, 0.02% (w/v) SDS, and 20% (v/v) methanol using electrophoresis. Subsequently, membranes were incubated for 1 h in blocking buffer containing 5% (w/v) fat-free milk powder and 1% (w/v) bovine serum albumin (BSA) in TRIS-buffered saline (TBS) supplemented with 0.1% (v/v) Tween-20 (TBS-T). Membranes were then incubated overnight with primary antibodies diluted 1:2000 in blocking buffer. The primary antibodies were specific for subunit A of the V-ATPase and the V-PPase (AS09 467 and AS09-465, respectively, Agrisera, Sweden). After washing the membranes three times with TBS-T, the membranes were incubated with the secondary antibody for 45 min. Following washing again, immune-detection was done using the ECL chemiluminescent detection kit (Amersham). Films were then scanned and the relative intensity of bands was determined by Scion Image.

### Preparation of Tonoplast-Enriched Membrane Vesicles

Tonoplast vesicles were isolated from leaves of 4-week-old potato plants by combining differential and sucrose gradient centrifugations as was shown to be effective for potato tissue (Queiros et al., 2009). Total leaf tissue, without stems or petioles (around 100 g) was collected from 4-week-old potato plants (cv. Desiree and Mozart) grown on hydroponics and subjected to 0 and 60 mM NaCl for 1 week. The entire isolation procedure was performed at 4°C and at all intermediate steps the samples were kept on ice. Leaves of each cultivar and each treatment were ground separately by repeated transient pulsing in a blender containing 200 ml ice-cold extraction buffer. The extraction buffer consisted of 250 mM sucrose, 2 mM EDTA (pH 8.0), 2 mM dithiothreitol (DTT), 1 mM phenylmethyl sulphonyl fluoride (PMSF), 70 mM Tris–HCl (pH 8.0), 3 mM MgCl_2_, 100 mM KCl, 0.1% (w/v) BSA, 0.2% (w/v) polyvinylpolypyrrolidone (PVPP), and 10 μM cantharidin (a phosphatase inhibitor). The homogenate was filtered through four layers of miracloth and centrifuged at 10,000 *g* for 10 min. The supernatants were centrifuged at 100,000 *g* for 1 h. The microsomal pellet was resuspended in 10 ml ice-cold resuspension buffer containing 15% (v/v) glycerol, 1 mM DTT, 1 mM EDTA (pH 7.5), 1 mM PMSF, 20 mM Tris–HCl (pH 7.5), and 10 μM cantharidin. The diluted microsomal fraction was separated in tonoplast membrane-enriched vesicles and plasma membrane-enriched vesicles by 3 h centrifugation at 80,000 *g* (Beckman SW 28 rotor) on a 0–32–46% (w/v) sucrose step gradient. Next to sucrose, the step gradient contained 20 mM Tris–HCl (pH 7.5), 1 mM EDTA (pH 7.5), 1 mM DTT, 1 mM PMSF, and 10 μM cantharidin. Subsequently, the tonoplast-enriched vesicles were collected at the 0–32% sucrose interface and approximately four times diluted in resuspension buffer and centrifuged at 100,000 *g* for 30 min. The pellets were resuspended in 1 ml resuspension buffer. The tonoplast vesicles were homogenized by gently swirling before aliquoting. Aliquots were snap-frozen in liquid nitrogen and stored at -80°C until further use. Before freezing, the protein concentration was measured by means of a Bradford assay (Bio-Rad), using BSA as a protein standard.

### Fluorescence Quench Assays

Tonoplast vesicles were used in proton transport assays. H^+^-transport across the tonoplast vesicles was energized by the addition of ATP or PPi in different concentrations as indicated in the “Results” section. Prior to measuring the substrate-dependent proton pump activity, the vesicles were characterized for their purity as contamination with vesicles derived from the plasma membrane is possible. This was done by adding 1 mM ATP to energize ATPases (both the V-ATPases and the PM-ATPases if present) in the presence of several concentrations of bafilomycin. Bafilomycin is a specific inhibitor of the activity of the V-H^+^-ATPase, and does not affect the activity of the P-type H^+^-ATPase found in plasma membranes or any other H^+^-ATPase. Therefore, a strong inhibition of ATP-dependent H^+^ pumping activity by bafilomycin is an indication that isolated vesicles are enriched in tonoplast membranes. The initial percentage rate of the fluorescence change of 2 μM 9-amino-6-chloro-2-methoxyacridine (ACMA; Δ%F min^-1^ mg^-1^ protein) was determined. The reaction medium contained 10 mM Mops-TRIS (pH 7.0), 5 mM MgCl_2_ and 100 mM KCl and 20 μg of tonoplast vesicles and fluorescence quenching was monitored using a spectrophotometer (Hitachi) at excitation and emission wavelengths of 415 and 485 nm, respectively, as described by Queiros et al. (2009). A pH gradient was established by activation of the V-H^+^-PPase with 0.5 mM PPi in reaction media as described above. After the fluorescence had reached a steady state, the reaction medium was supplemented with different concentrations of Na_2_SO_4_ as indicated in the “Results” section to dissipate the pH gradient. The initial slope of the recovery of fluorescence quench represented the activity of Na^+^/H^+^ antiport activity, expressed as Δ%F min^-1^ mg protein^-1^.

### Statistics

All data reported in this work were obtained from three or four biologically independent pot experiments as indicated in the figures. Each pot experiment was arranged in a randomized design with two salt treatments. Data were analyzed with one-way ANOVA and two-way ANOVA with a multiple comparison test (Tukey’s test; SPSS) or Student’s *t*-test as indicated in the figures. Differences between treatments and/or cultivars were considered significant when *P* < 0.05.

## Results

### Na^+^ and K^+^ Concentrations, Water Status and Osmolality of the Leaves

**FIGURE 1:**
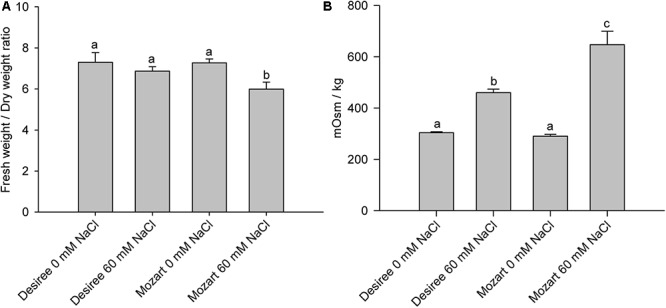
Leaf parameters of Desiree and Mozart grown without and with 60 mM NaCl. **(A)** The fresh weight/dry weight ratio for each treatment and cultivar. **(B)** The osmolality (mOsm kg^-1^) of the leaf sap of each treatment and cultivar. The osmolality of the leaf sap was measured with an osmometer (Wescor 5500, Wescor, Logan, UT, United States). Results shown are the average of three independent experiments ± SEM. Different letters indicate a statistically significant treatment effect (one-way ANOVA; *P* < 0.05). A two-way ANOVA showed a treatment × cultivar interaction effect for the fresh weight/dry weight ratio and the osmolality (*P* < 0.05).

**Table 1** shows Na^+^ and K^+^ concentrations accumulated in leaf tissue after salt treatment. Mozart accumulated over 30% more Na^+^ in leaf tissue compared to Desiree. The K^+^ concentrations in leaves of Desiree grown at 60 mM NaCl was the same as that of the control leaves, whereas leaves of Mozart grown at 60 mM NaCl showed a significant reduction in K^+^ concentrations by more than 40%. Subsequently, we determined the fresh weight/dry weight (FW/DW) ratio of leaves and the osmolality of the leaf sap (**Figure 1**) as described in the “Materials and Methods” section. Leaves collected from salt-treated plants of Mozart showed a significantly lower FW/DW ratio as compared to leaves collected from salt-treated plants of Desiree and the control plants (**Figure 1A**). The lower water uptake observed in Mozart leaves treated with NaCl, combined with the higher Na^+^ concentrations in the leaves resulted in a higher osmolality of leaf sap (**Figure 1B**). Salt treatment increased the leaf sap osmolality of both salt-treated cultivars significantly, but the osmolality was about 25% higher in Mozart after salt treatment as compared to that of Desiree (**Figure 1B**). Next, we addressed the question whether the observed differences in Na^+^ and K^+^ accumulation relate to differences in the activity of vacuolar transporters.

**Table 1:** Na^+^ and K^+^ concentrations in the leaves of the potato cultivars Desiree and Mozart grown in the absence and presence of 60 mM NaCl.

Cultivar	Treatment	[Na^+^] mg g^-1^ DW	[K^+^] mg g^-1^ DW
Desiree	0 mM NaCl 60 mM NaCl	2.2 ± 0.3 (a) 17.8 ± 1 (b)	34.6 ± 2.9 (a) 31.4 ± 3.3 (a)
Mozart	0 mM NaCl 60 mM NaCl	2.2 ± 0.2 (a) 24.2 ± 1.8 (c)	43.7 ± 4.4 (a) 25.4 ± 2.7 (b)

*Results are the mean ± SEM for four biological replicates. Different letters indicate a statistically significant treatment effect (one-way ANOVA; *P* < 0.05). A statistically significant treatment × cultivar interaction effect was found for both Na^+^ and K^+^ concentrations (two-way ANOVA; *P* < 0.05). DW, plant dry weight*.

### Proton Pump Activity in Isolated Vesicles Is Bafilomycin Sensitive

**FIGURE 2:**
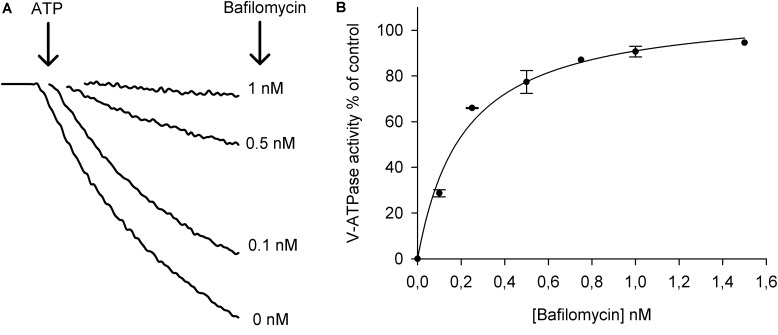
Activation of the V-H^+^-ATPase in tonoplast vesicles isolated from leaves and the sensitivity for the inhibitor bafilomycin. **(A)** ATP-driven fluorescence quench of ACMA in the absence of bafilomycin (0 nM) and in the presence of 0.1, 0.5, and 1 nM bafilomycin. The reactions were started by the addition of 1 mM Mg ATP to the assay medium containing 20 μg protein of tonoplast vesicles. **(B)** Dose response curve of the inhibition of V-H^+^-ATPase activity by bafilomycin. The curve was fitted to a Michaelis–Menten equation with the aid of SigmaPlot software package (*n* = 3 ± SEM).

**Figure 2A** shows that increasing concentrations of bafilomycin recovered the ATP-driven fluorescence quench. With the initial fluorescence quenches measured without bafilomycin set to 100%, it was found that the EC_50_ of the bafilomycin concentration was as low as 0.2 nM for ATP-dependent pump activity and with 1.5 nM bafilomycin in the reaction medium the ATP-dependent pump activity was inhibited by around 97% (**Figure 2B**). These results indicate that the isolated membrane vesicles are highly enriched in vacuolar membranes.

### Effect of Salt Treatment on V-H^+^-PPase- and V-H^+^-ATPase Activity

**FIGURE 3:**
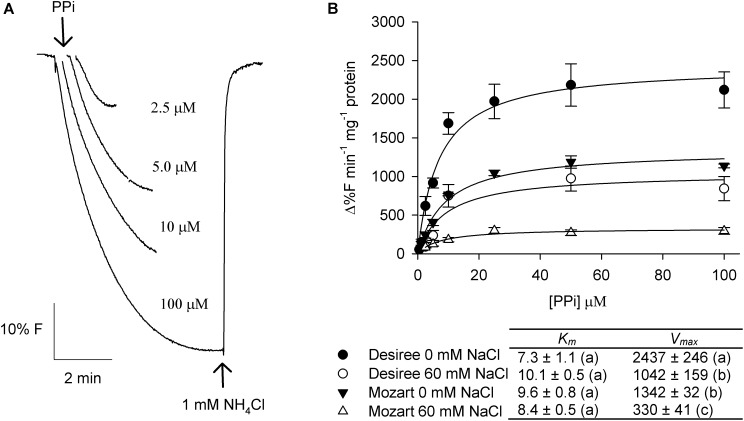
Effect of salt stress on the PPi-dependent proton transport activity in tonoplast vesicles isolated from potato leaves. **(A)** Representative traces of proton pump activity in tonoplast vesicles isolated from leaves of the cultivar Desiree. The V-H^+^-PPase was activated by the addition of increasing concentrations of PPi to the assay medium containing 20 μg protein of membrane vesicles. After equilibration 1 mM NH_4_Cl was added to release the quench. **(B)** Dose response curves of the initial rates of proton pump activity of the V-H^+^-PPase in dependence of the substrate PPi concentration for both cultivars treated with and without 60 mM NaCl. The curves were fitted with the Michaelis–Menten equation with the aid of SigmaPlot software package. The average *K*_m_ and *V*_max_ values as shown in the table were calculated from the fit of each independent experiment (*n* = 3 ± SEM). Different letters indicate a statistically significant treatment effect (one-way ANOVA; *P* < 0.05). A two-way ANOVA showed a treatment × cultivar interaction effect for *V*_max_ (*P* < 0.05).

**FIGURE 4:**
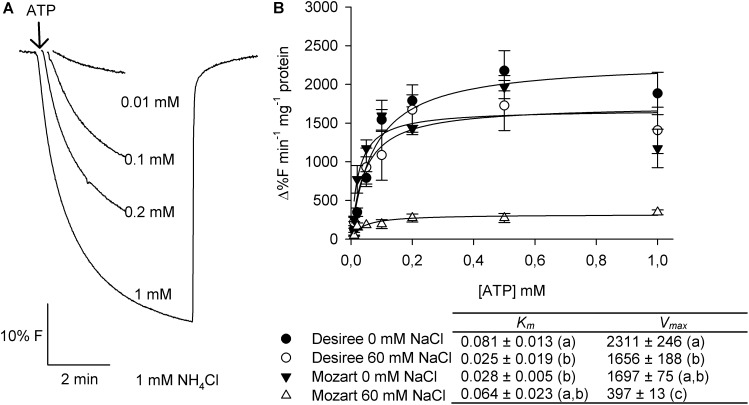
Effect of salt stress on the ATP-dependent proton transport activity in tonoplast vesicles isolated from potato leaves. **(A)** Representative traces of proton pump activity in tonoplast vesicles isolated from leaves of the cultivar Desiree. The V-H^+^-ATPase was activated by the addition of increasing concentrations of ATP to the assay medium containing 20 μg protein of membrane vesicles. After equilibration of the quench, 1 mM NH_4_Cl was added to release the quench. **(B)** Dose response curves of the initial rates of proton pump activity of the V-H^+^-ATPase in dependence of the substrate ATP concentration for both cultivars treated with and without 60 mM NaCl. The curves were fitted with the Michaelis–Menten equation with the aid of SigmaPlot software package. The average *K*_m_ and *V*_max_ values as shown in the table were calculated from the fit of each independent experiment (*n* = 3 ± SEM). Different letters indicate a statistically significant treatment effect (one-way ANOVA; *P* < 0.05). A two-way ANOVA showed a treatment × cultivar interaction effect for both *K*_m_ and *V*_max_ (*P*< 0.05).

The substrate-induced quench appeared almost instantaneously upon energization and after signal stabilization, the addition of NH_4_Cl rapidly dissipated the pH gradient (**Figure 3A** for PPi and **Figure 4A** for ATP). Measurements were done with vesicles derived from both cultivars treated with 0 and 60 mM NaCl and the initial rates of proton pumping were plotted against increasing concentrations of PPi and ATP (**Figures 3B**, **4B**). The curves showed Michaelis–Menten-like kinetics and both kinetic parameters, *K*_m_ and *V*_max_, were calculated and averaged, where *K*_m_ represents the affinity for the substrate and *V*_max_ represents the maximum pump activity.

The *K*_m_ of PP_i_-dependent proton pumping was similar for both cultivars and was not modulated by salt (**Figure 3**). The V-H^+^-PPase activity in vesicles obtained from Desiree was significantly higher, in control conditions (twofold) and after salt treatment (threefold), compared to the V-H^+^-PPase activity from Mozart (**Figure 3**). Furthermore, salt treatment had a larger negative effect on the V-H^+^-PPase activity in Mozart (-75%) as compared to Desiree (-55%; **Figure 3**).

The V-H^+^-ATPase activity in control plants of Mozart showed a significant lower *K*_m_ value (0.028 mM) compared to control plants from Desiree (0.081 mM; **Figure 4**). After salt treatment, the *K*_m_ value significantly decreased for Desiree (∼70%) but not for Mozart (**Figure 4**). The V-H^+^-ATPase activity was significantly lower in Mozart plants after salt treatment (-80%) while the V-H^+^-ATPase activity in Desiree was only 30% lower after salt treatment (**Figure 4**). The V-H^+^-ATPase activity of salt-treated Desiree plants was similar to that of control plants of Mozart (**Figure 4**).

### Immunological Detection of the V-ATPase A-Subunit and the V-PPase in Response to Salt

**FIGURE 5:**
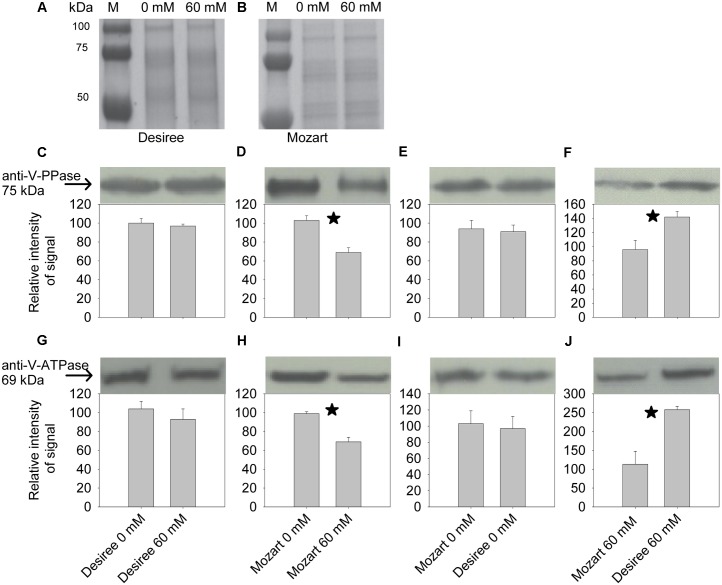
Electrophoretic polypeptide pattern and Western blot analysis of tonoplast proteins from the potato cultivars Desiree and Mozart. Tonoplast vesicles were isolated from leaves of plants grown in the absence and presence of NaCl as indicated in the figure by 0 and 60 mM, respectively. **(A,B)** Coomassie blue stained gel of tonoplast proteins from Desiree and Mozart, respectively. Lane M represents the protein marker. Western blots **(C–J)** were scanned into gray scale and the optical density was quantified by Scion Image. Blots were probed with anti-V-PPase **(C–F)** and anti-V-ATPase (A-subunit) **(G–J)** of mung bean. Panels **(C,G,D,H)** show the effect of salt treatment on the amount of V-H^+^-ATPase and the V-H^+^-PPase in both cultivars. Panels **(E,I,F,J)** show the effect of the cultivar on the amount of V-H^+^-ATPase and the V-H^+^-PPase in both salt treatments. The molecular masses of the immunostained polypeptides are indicated (arrows). Values represent mean ± SEM of four independent replicates. Asterisks indicate a statistically significant treatment effect (Student’s *t*-test; *P* < 0.05).

Western blotting shows a single band at 75 kDa band for the V-H^+^-PPase (**Figures 5C–F**) and a 69-kDa band for the V-H^+^-ATPase subunit A (**Figures 5G–J**). Densitometric analysis of the relative abundance of the V-H^+^-PPase showed that salt treatment had no effect on the V-H^+^-PPase amount in Desiree (**Figure 5C**), whereas in Mozart the amount of V-H^+^-PPase significantly decreased more than 30% (**Figure 5D**). These changes in protein amount only partly explain the observed salt-induced reductions in the V-H^+^-PPase activity (**Figure 3B**), with a twofold and threefold reduction in Desiree and Mozart, respectively. Furthermore, the V-H^+^-PPase protein amounts in control plants of Desiree and Mozart were equal (**Figure 5E**) and did not correlate with the differences in V-H^+^-PPase activity in control plants (45% lower in Mozart as compared to Desiree; **Figure 3B**).

Analysis of the V-H^+^-ATPase Western blots showed a pattern similar to that of the V-H^+^-PPase: no salt-induced change in Desiree and a significant 40% reduction of the V-H^+^-ATPase in Mozart (**Figures 5G,H**).

### Vacuolar Na^+^/H^+^ Antiport Activity and Response to Salt

**FIGURE 6:**
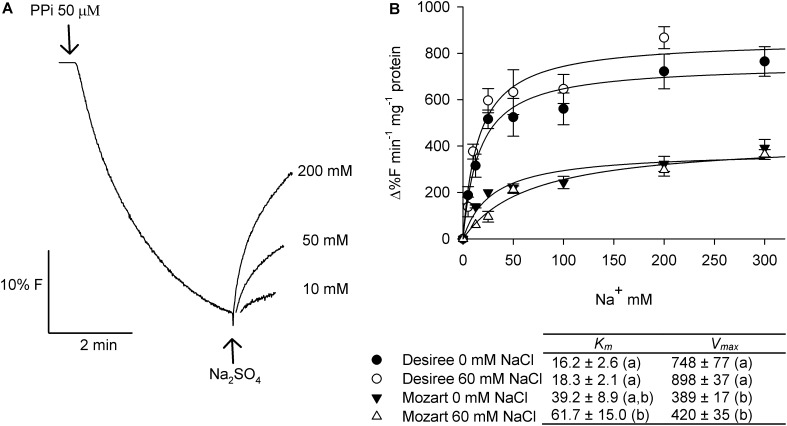
Effect of salt stress on Na^+^/H^+^ antiport activity in tonoplast vesicles isolated from potato leaves. **(A)** Representative curve showing the dissipation of the PPi-generated fluorescence quench (H^+^ gradient) with the addition of increasing concentrations of Na^+^ to tonoplast vesicles from the cultivar Desiree. **(B)** Dose response curves where the initial rates of Na^+^-induced proton gradient dissipation are plotted against the Na^+^ concentration (2.5–150 mM Na_2_SO_4_). Tonoplast vesicles were isolated from leaves of Desiree and Mozart plants grown in the absence and the presence of 60 mM NaCl. Experimental data of each individual curve (*n* = 3 ± SEM) were fitted to the Michaelis–Menten equation with the aid of SigmaPlot software package and the average *K*_m_ and *V*_max_ values are shown in the table. Different letters indicate a statistically significant treatment effect (one-way ANOVA; *P* < 0.05). A statistically significant treatment × cultivar interaction effect was found for both *K*_m_ and *V*_max_ (two-way ANOVA; *P* < 0.05).

**Figure 6A** shows the effect of different concentrations of Na^+^ on the dissipation of a pre-established pH gradient in tonoplast vesicles from control- and salt-treated plants of both cultivars. The ACMA fluorescence recovered with the addition of increasing concentrations of Na^+^ and followed saturation kinetics. Kinetic analysis of the saturation curves revealed that the salt treatment hardly affected the *V*_max_ of Na^+^ transport in vesicles derived from either Desiree or Mozart (**Figure 6B**). However, the *V*_max_ for Na^+^/H^+^-antiport was significantly higher (twofold) in Desiree as compared to the *V*_max_ from Mozart. Furthermore, the Na^+^/H^+^ antiport system of Desiree showed a twofold to threefold higher affinity for Na^+^ than the Na^+^/H^+^ antiport system of Mozart (**Figure 6B**).

### Gene Expression of Vacuolar Ion Transporters

**FIGURE 7:**
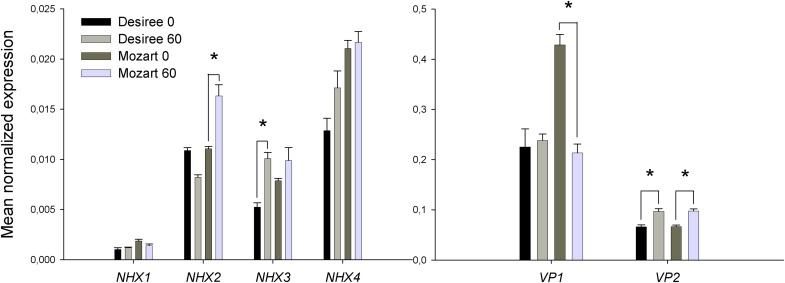
Expression of potato gene transcripts with homology to isoforms of *SlVP1* and *SlVP2* (*VP1* and *VP2*) and with homology to isoforms of *SlNHX1*-*SlNHX4* (*NHX1*–*NHX4*). The expression of the transcripts was analyzed by qPCR in leaf tissue, as described in the Section “Materials and Methods” in the cultivars Mozart and Desiree grown in the absence and presence of 60 mM NaCl. Expression levels for all gene transcripts are normalized to the expression of the potato elongation factor *ef1α*. Data are means ± SEM of three independent experiments. One-way ANOVA and two-way ANOVA were performed and asterisks indicate a statistically significant treatment × cultivar interaction effect.

Three potato unigenes were found for three *V-PPases* corresponding to two type I and one type II V-H^+^-PPases as previously described for tomato (Mohammed et al., 2012). Type I V-H^+^-PPases depend on cytosolic K^+^ for their activity and are localized in the tonoplast whereas type II V-H^+^-PPases are K^+^ insensitive and directed to the Golgi apparatus (Maeshima, 2000). Unigenes of the two type I V-H^+^-PPases (*SlVP1* and *SlVP2*) were used for expression analysis (**Table 2**). A homology search of four *SlNHX* isoforms from tomato (Gálvez et al., 2012) on the public database SOL Genomics (see footnote 1) resulted in three unigenes with homology to *SlNHX2*, *SlNHX3*, and *SlNHX4*, respectively. A homolog of *SlNHX1* was absent on the SOL Genomics network but the genomic sequence was found in the public database of the Potato Genome Sequencing Consortium^3^ (Database file: PGSC_DM_v4.03) and primers were designed in putative exons (**Table 2**). Within the four *NHX* isoforms tested, the expression level of *StNHX1* was overall low, those of *StNHX4* were highest and expression levels of *StNHX2* and *StNHX3* were intermediate (**Figure 7** and Supplementary Data Sheet 1). Salt treatment induced *StNHX2* transcripts in Mozart and induced *StNHX3* transcripts in Desiree and no effect of salt was found on *StNHX4* transcripts (**Figure 7**).

**Table 2:** Homologs of a selection of genes involved in vacuolar ion transport were identified in potato by querying a database containing potato SGN unigenes (see footnote 1) and genomic sequences (see footnote 2) using coding sequences from tomato in blast searches.

Gene	Annotation/Gene accession	% Homology to	Primer 5′→3′	Amplicon size
*SlVP1*	AB300442	97% to SGN-U268918	Fw. CTCTTCCCCCGTATTTCACA Rv. CCTCCACAGCCTAACACCAT	100 bp
*SlVP2*	LES278019	97% to SGN-U269308	Fw. GGTCCTATCAGTGACAATGCTG Rv. AACAGAGCCAGGGACACAAG	161bp
*SlNHX1*	AJ306630	97% to See text	Fw. TGTTGATCCCTTTCGACCAT Rv. CCAAATAGGGGTCGCATAAA	111 bp
*SlNHX2*	AJ306631	98% to SGN-U296226	Fw. TTGGCACAGACGTGAACCTA Rv. GTGGCTTCTGACCAGTGACA	107 bp
*SlNHX3*	AM261867	98% to SGN-U273140	Fw. AGAAGTCTCCGGAGGAGAGG Rv. GCGTTGGCACGTAACTGAGTA	139 bp
*SlNHX4*	AM261867	98% to SGN-U275721	Fw. TCCTAGGCAGTCGAGAGGAC Rv. TGGTGCGGTAAGTAGCATCC	89 bp

Expression of *StVP1* remained unchanged in salt-treated plants of Desiree, whereas in Mozart the expression level in control plants was twofold higher as in Desiree, but salt stress reduced the expression twofold (**Figure 7**). Expression of *StVP2* was lower than that of *StVP1* and a small significant increase in expression was observed in both cultivars upon salt stress (**Figure 7**).

## Discussion

### General

The study presented here confirmed our previous results that Mozart accumulates more Na^+^ in leaves (35% more) as compared to salt-treated Desiree plants (**Table 1**) (Jaarsma et al., 2013). If Mozart was a salt includer with an efficient vacuolar Na^+^ sequestration mechanism, then it might profit from the higher Na^+^ levels by using Na^+^ as osmoticum (Shabala and Mackay, 2011). However, the lower FW/DW ratio of leaves of salt-treated Mozart plants (**Figure 1A**) indicates that these plants have difficulties to take up water in their leaves resulting in higher leaf sap osmolality (**Figure 1B**). In view of the premature senescence and the sensitivity of leaf growth of Mozart to salt stress found in our previous study, we hypothesized before that the vacuolar sequestration mechanism operates far from optimal in Mozart (Jaarsma et al., 2013).

### Generation of the PMF Is Modulated by Salt and Is Cultivar-Dependent in Potato

In this study, we used ACMA fluorescence quench assays and the V-H^+^ATPase and the V-H^+^-PPase generated and maintained the electrochemical gradient across the vacuolar membrane in both cultivars, as was found before for potato tissue (Queiros et al., 2009). In both cultivars, the V-H^+^-PPase activity showed a major salt-induced reduction, but the reduction was larger in Mozart than in Desiree (**Figure 3**). Furthermore, the V-H^+^-PPase activity in control plants of Desiree was already higher than the V-H^+^-PPase activity in control plants of Mozart (**Figure 3**). From these results, we conclude that the V-H^+^-PPase is salt sensitive in both cultivars and that V-H^+^-PPase activity is cultivar dependent and higher in Desiree.

The V-H^+^-ATPase activity showed a salt-induced reduction of ∼80% in Mozart, whereas salt treatment reduced the V-H^+^-ATPase activity in Desiree by ∼30% (**Figure 4**). Overall, salt considerably reduced the activity of both proton pumps in vesicles isolated from Mozart as compared to those of Desiree. As a consequence, Desiree generates a higher PMF across the tonoplast that can be used to energize the vacuolar transport of Na^+^.

### V-H^+^-PPase and V-H^+^-ATPase Protein Amounts Show a Salt-Induced Decrease in Mozart

The Western blots showed a salt-induced reduction in the amount of V-H^+^-PPase proteins in salt-treated plants of the cultivar Mozart (-40%; **Figure 5**) and this correlated reasonably well with the salt-reduced pump activity observed in Mozart. The transcript levels of the *StVP1* gene were twofold lower in salt-treated Mozart plants as compared to control plants (**Figure 7**). These lower transcripts correlated to the salt-induced reduction in the protein amounts found for V-H^+^-PPase. So, for Mozart, the salt-reduced pump activity may be a result of the reduction in gene expression and total protein amount. However, there was no reduction in the amounts of the V-H^+^-PPase protein in Desiree after salt treatment (**Figure 5**). Hence, the correlation found for Mozart between lower V-H^+^-PPase protein amounts and lower V-H^+^-PPase activity does not hold for Desiree.

Furthermore, a reduction was found for the amount of V-H^+^-ATPase proteins in salt-treated plants of Mozart but not in salt-treated plants of Desiree (**Figure 5**). To measure the protein amounts of the V-H^+^-ATPase, we used antibodies to detect subunit A (see section “Materials and Methods” section). Subunit A is located in the cytosolic V_1_ domain of the V-H^+^-ATPase protein and subunits located in the more stable V_0_ complex of the V-H^+^-ATPase protein may be considered for measurements of protein amounts. For example, Baisakh et al. (2012) expressed subunit c1 from a halophyte grass *Spartina alterniflora* in rice and the *SaVHAc1* expressing plants showed enhanced tolerance to salt stress. Furthermore, after salt treatment, transcript levels of subunit c increased earlier than transcript levels of subunit A or B did in *Mesembryanthemum crystallinum* (Low et al., 1996). So, analysis of other V-H^+^-ATPase subunits would further contribute to elucidate the differences of V-H^+^-ATPase activity between Mozart and Desiree.

### Substrate-Dependent Vacuolar H^+^ Transport Shows Varying Results Among Species and Tissues

The effect of salt on vacuolar ATP- and PPi-dependent H^+^-transport has been extensively studied in the last decades for many plant species, with varying results. One study with rice callus lines differing in salt tolerance showed a strong increase in vacuolar pump activity when salt-treated and more so in the salt tolerant lines (Pons et al., 2011). The halophytes *M. crystallinum* and *Suaeda salsa* showed a salt-dependent increase in V-H^+^ATPase activity (Barkla et al., 1995; Qiu et al., 2007), while in *Cucumis sativus* ATP- and PPi-dependent proton transport decreased in roots after 50 mM NaCl treatment (Kabala and Klobus, 2008). In tonoplast vesicles derived from roots of the moderate salt tolerant sunflower (*Helianthus annuus*), ATP- and PPi-dependent proton transport increased in plants treated with 150 mM NaCl (Ballesteros et al., 1996). Salt treatment induced V-H^+^-ATPase and V-H^+^-PPase activity in vesicles derived from *Spinacia oleracea*, whereas V-H^+^-ATPase and V-H^+^-PPase activity in *Salicornia dolichostachya* was not affected by salt treatment (Katschnig et al., 2014). These different and often contrasting results suggest major differences between species and tissues in the capacity to control vacuolar proton pump activity. It should be noted that the severity and length of the salt treatments varied among the above-mentioned studies, what may as well be a cause for variation in the results.

However, our results showed that in leaves of potato plants, vacuolar ATP- and PPi-dependent H^+^ transport decreased after salt treatment (**Figures 3**, **4**), the amount of V-H^+^-PPases and V-H^+^-ATPases proteins decreased in Mozart but not in Desiree (**Figure 5**) and although transcripts of *StVP2* were low, expression was induced in both cultivars after salt treatment (**Figure 7**). These results resemble previous findings for roots of wheat showing that V-H^+^-ATPase-, and V-H^+^-PPase activity and V-H^+^-ATPase-, and V-PPase protein amount decreased, while expression of *TaVP1* and *TaVP2* increased upon salt stress (Wang et al., 2000, 2009). Recently, other functions besides generating a PMF for the V-H^+^PPases have been reviewed, such as increased auxin fluxes, increased nutrient uptake, or increased carbohydrate metabolism (Schilling et al., 2017) and these functions may also contribute to growth during salt conditions.

### Activity of Na^+^/H^+^ Antiport and *NHX* Expression in Response to Salt

NHXs function in cellular Na^+^-, K^+^-homeostasis and are either targeted to the vacuole (Class I) or targeted to the pre-vacuoles (Class II) (Rodríguez-Rosales et al., 2009). Expression of multiple *NHX* isoforms of both classes in a variety of plant species improves salt tolerance (Rodríguez-Rosales et al., 2009; Agarwal et al., 2012) although this may not simply be due to enhanced Na^+^ accumulation into the central vacuole since NHX transporters show varying degrees of Na^+^/H^+^ and K^+^/H^+^ antiport activity (Barragan et al., 2012; Zhang et al., 2015). For example, SlNHX2 confers K^+^/H^+^ exchange and localizes to pre-vacuolar vesicles, but tomato plants with enhanced *SlNHX2* expression and the tomato *nhx2* null mutants were more- and less-salt tolerant, respectively (Rodríguez-Rosales et al., 2008). In addition, a *nhx4* null mutation in Arabidopsis showed enhanced tolerance to salt stress and lower Na^+^ and higher K^+^ levels than wild-type plants (Li et al., 2009).

The four *StNHX* transcripts analyzed in this study are homologs of the better characterized *SlNHX* genes from tomato and the relative expression of the four isoforms in leaves of non-stressed tomato (Gálvez et al., 2012) and potato plants (**Figure 7**) is similar: *SlNHX1* and *SlNHX4* transcript levels are relatively low and high, respectively, and those of *SlNHX2* and *SlNHX3* are intermediate. In our study, salt had no effect on *StNHX1* expression of either cultivar, induced the expression of *StNHX2* in Mozart, induced *StNHX3* in Desiree and no effect of salt was found on expression of *StNHX4* (**Figure 7**). It is difficult to translate changes in transcripts of a specific gene to changes in protein, let alone protein activity. Though, the relative moderate changes in expression of the *NHX* genes by salt are in line with our observation that the *V*_max_ of the Na^+^/H^+^ antiport activity is not different between control and salt-treated plants (**Figure 6**) and this low activity may explain the general moderate salt tolerance found for potato. However, what is different when comparing the two cultivars and not in line with the *NHX* expression data is that the *V*_max_ for Na^+^/H^+^ antiport activity is twofold higher in Desiree and the *K*_m_ for Na^+^ is at least twofold lower in Desiree (**Figure 6**).

Higher NHXs abundance in Desiree and/or post-translational regulation of NHXs in Desiree may cause the observed differences in antiport activity and affinity for Na^+^. It has been proposed that protein–protein interaction, phosphorylation, and/or glycosylation of the C-terminus can differentially regulate the antiport activity of NHXs (Qiu et al., 2004; Bassil et al., 2012; Bassil and Blumwald, 2014). So far, a calmodulin-like protein (AtCaM15) was found to bind the C-terminus of AtNHX1 within the vacuolar lumen in yeast cells and modify the cation selectivity in a Ca^2+^ and pH-dependent manner for either K^+^ or Na^+^ (Yamaguchi et al., 2005). The authors suggested that in normal physiological conditions (high free vacuolar Ca^2+^ and pH ∼5.5) AtCam15 is bound to AtNHX1 rendering the antiporter with a higher affinity for K^+^ over Na^+^. Higher cytosolic Na^+^ concentrations may lead to vacuolar alkalization and subsequently to the dissociation of AtCam15 from AtNHX1 resulting in a higher affinity for Na^+^ over K^+^ (Yamaguchi et al., 2005).

Different phosphorylation sites in the C-terminus of NHX proteins appear to be present in many sequenced plant species so far, but await further functional analysis (Bassil et al., 2012). For example, sequencing the *NHXs* and homologs of *AtCaM15* from both potato cultivars may elucidate differences in these regulatory sites and reveal clues about the overall lower Na^+^/H^+^ antiport activity found for Mozart.

## Author Contributions

RJ and AdB designed the experiments. RJ conducted the experiments. RJ and AdB wrote the manuscript.

## Conflict of Interest Statement

The authors declare that the research was conducted in the absence of any commercial or financial relationships that could be construed as a potential conflict of interest.

## Footnotes

1 http://solgenomics.net

2 http://solanaceae.plantbiology.msu.edu/pgsc_download.shtml

3 http://solanaceae.plantbiology.msu.edu/pgsc_download.shtml
